# Automatic Generation of Object Shapes With Desired Affordances Using Voxelgrid Representation

**DOI:** 10.3389/fnbot.2020.00022

**Published:** 2020-05-14

**Authors:** Mihai Andries, Atabak Dehban, José Santos-Victor

**Affiliations:** ^1^Institute for Systems and Robotics (ISR/IST), LARSyS, Instituto Superior Técnico, Universidade de Lisboa, Lisbon, Portugal; ^2^Université de Lorraine, CNRS, Inria, LORIA, Nancy, France; ^3^Champalimaud Centre for the Unknown, Lisbon, Portugal

**Keywords:** affordance, generative design, computer aided design (CAD) algorithms, voxel grids, shape generation, affordance testing

## Abstract

3D objects (artifacts) are made to fulfill functions. Designing an object often starts with defining a list of functionalities or affordances (action possibilities) that it should provide, known as *functional requirements*. Today, designing 3D object models is still a slow and difficult activity, with few Computer-Aided Design (CAD) tools capable to explore the design solution space. The purpose of this study is to explore shape generation conditioned on desired affordances. We introduce an algorithm for generating voxelgrid object shapes which afford the desired functionalities. We follow the principle *form follows function*, and assume that object forms are related to affordances they provide (their functions). First, we use an artificial neural network to learn a function-to-form mapping from a dataset of affordance-labeled objects. Then, we combine forms providing one or more desired affordances, generating an object shape expected to provide all of them. Finally, we verify in simulation whether the generated object indeed possesses the desired affordances, by defining and executing affordance tests on it. Examples are provided using the affordances contain-ability, sit-ability, and support-ability.

## 1. Motivation

**Figure 1:**
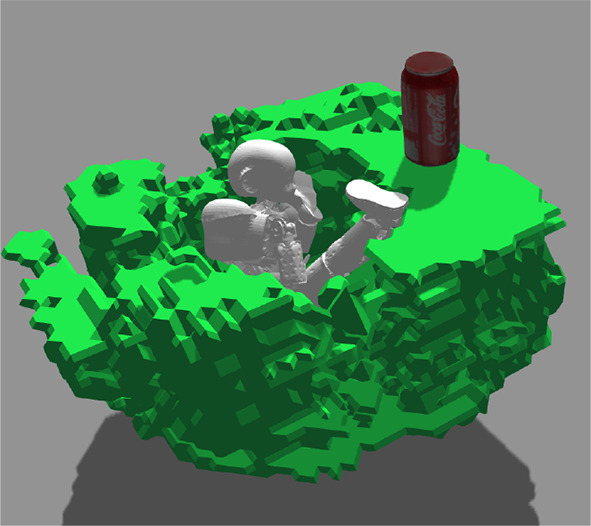
Generated object shape providing the *contain-ability* and *support-ability* affordances.

**Figure 2:**
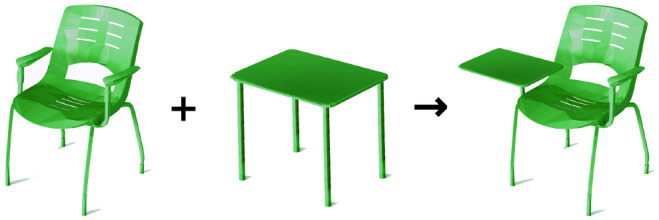
Illustration of the affordance combination concept: combining the sit- and lean-abilities of chairs with the support-ability of tables, to obtain a hybrid object providing abilities to sit, lean, and support.

Design cycles of products are lengthy, as they usually involve thousands of decisions on the form of the product that will implement the desired functionalities/affordances. Despite efforts in the last two decades to accelerate the workflow using CAD techniques (Kurtoglu, 2007; Autodesk Inc., 2017), most of the design process is still done manually. In an attempt to solve this pertinent problem, the Defense Advanced Research Projects Agency (DARPA) launched in 2017 the *Fundamental Design* call for research projects on conceptual design of mechanical systems, that would enable the generation of novel design configurations (DARPA, 2017). The purpose of our study is to explore automatic shape generation conditioned on desired affordances, as illustrated in Figures 1, 2.

This paper also has another motivation stemming from robotics. Traditionally, research in autonomous robots deals with the problem of recognizing affordances of objects in the environment: i.e., given an object, what actions does it afford to do? This paper addresses the inverse problem: given some affordances, what shape would provide them?

**Figure 3:**
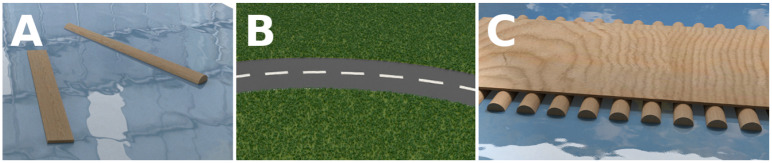
Illustration of the affordance combination concept: the features that describe wooden beams and flat roads can be combined, to obtain an object design that possesses both *float-ability* and *traverse-ability*: a pontoon bridge. **(A)** *Wooden* beams have the *float-ability* affordance. **(B)** *Flat* roads have the *traverse-ability* affordance. **(C)** *Flat wooden* roads offer both *float-ability* and *traverse-ability*.

This paper presents a method for automatic generation of object voxelgrid shapes with desired affordances. It does so by automatically relating object forms to their affordances, and then applying this knowledge to conceive new object forms that satisfy given functional requirements. In a sense, this method performs *affordance arithmetic*—by analogy with *shape arithmetic* (Wu et al., 2016)—through manipulation of latent vectors corresponding to affordances (as opposed to shapes). Figures 2, 3 illustrate the concept of combining *features* describing two different objects to create another object possessing the *affordances* of both initial objects. A second contribution is the introduction of experiments to verify the presence of affordances in generated object shapes using a physics simulator—both by defining explicit tests in the simulator, and by using state-of-art affordance detectors. To summarize, this paper presents a novel method for extracting and combining forms into novel objects with desired affordances, and introduces tests for objectively checking the presence of affordances.

The remainder of the paper is organized as follows. Section 2 presents an overview of related work in object design, shape descriptors, and object affordances. Section 3 describes our methodology, detailing the envisioned workflow for using this technology. It also describes operators for manipulating object forms. In section 4 we discuss obtained results and describe the drawbacks of the method. Finally, section 5 draws a conclusion and lists opportunities for future work.

## 2. Related Literature

The literature review is organized in three sections, detailing the state-of-the-art in the three fields at the intersection of which this study finds itself: object design, object shape descriptors (for manipulation of object forms), and learning of object affordances (for relating object forms to affordances).

### 2.1. Object Design

The idea of getting inspiration from previous designs when conceiving a new object is not new and in the literature it is referred to as *Analogical reasoning* or *Design reuse*. A standard practice in design is to consult *knowledge ontologies* (Bryant et al., 2005; Kurtoglu and Campbell, 2009; Bhatt et al., 2012) that contain function-to-form mappings (Umeda and Tomiyama, 1997; Funkhouser et al., 2004; Kurtoglu, 2007). However, the knowledge acquisition required to populate such ontologies involves a (non-automated) process known as *functional decomposition*, in which a human analyses existing objects by disassembling them into components and notes the functionality provided by each component. A related review on object functionality inference from shape information was presented by Hu et al. (2018).

Recently, generative design emerged as an automated technique for exploring the space of 3D object shapes (Bentley, 1996; Autodesk Inc., 2017, 2018). It formulates the shape search as an optimization problem, requiring an initial solution, a definition of parameters to optimize, and rules for exploring the search space. However, it is far from trivial to identify rules for intelligent exploration of the shape space, that would provide results in reasonable time. Mitra et al. (2014) presented a survey of structure-aware shape processing methods, specifically detailing the problems of segmenting object parts, parameterizing them, and identifying correlations between parts. In a similar context of generative design, Umetani (2017) employed an AutoEncoder to explore the space of car shapes.

### 2.2. Object Shape Descriptors

Object shape descriptions serve two purposes: (1) they contain object shape features, which are used to study the form-to-function relationship, and (2) they serve as basis for the reconstruction of 3D object models. State-of-the-art techniques for automatically extracting object descriptors are based on Neural Networks, typically Convolutional Neural Networks, which have superseded methods based on hand-crafted features.

Auto-Encoders (Girdhar et al., 2016) and Generative Adversarial Networks (Wu et al., 2016) are two popular approaches in generating 3D shapes from descriptions. These techniques learn a mapping from a low-dimensional (probabilistic) latent space to the space of 3D objects, allowing to explore the 3D object manifold. Recently, shape programs (Tian et al., 2019) were proposed for representing 3D object models composed of multiple parts, using programs that assemble parameterized shape primitives into a single object. It is a promising approach, generating high-level object descriptions (shape programs) which are easily interpretable by humans, as opposed to latent representations.

In this study, we used a Variational AutoEncoder (VAE) (Kingma and Welling, 2014; Rezende et al., 2014) to both extract features describing 3D objects, and reconstruct the 3D shape of an object when given such a description.

### 2.3. Object Affordances

A field of research that also focuses on linking object shapes with their functionality is that of *affordance learning*. It is based on the notion of *affordance* that defines an action that an object provides (or affords) to an agent (Gibson, 1979). In the context of this paper, we are interested in approaches that map object features to corresponding object affordances. The affordances that we study are the affordances in the habitat of humans, according to the formalism used by Baggs and Chemero (2020). In our use case (design for humans), we implicitly consider the agent to be human. Thus, we focus on affordances seen from a human perspective.

To detect affordances of objects, a common approach is to segment image regions (from RGB-D frames) with specific properties and tag them with corresponding affordance labels (Myers et al., 2015). An overview of machine learning approaches for detecting affordances of tools in 3D visual data is available in Ciocodeica (2016). Recent reviews on affordances in machine learning for cognitive robotics include Jamone et al. (2016), Min et al. (2016), and Zech et al. (2017).

This paper introduces a method to automatically learn shape descriptors and extract a form-to-function mapping, which is then employed to generate new objects with desired affordances, using a voxelgrid representation. The novelty lies in the extraction of *functional forms* and the use of *affordance arithmetic* (operations on object affordances) through manipulation of corresponding forms in a feature space. We thus apply the principle *form follows function* in an automated design setting, generating forms based on desired affordances. Following this principle, we assume that object forms are correlated to their affordances.

## 3. Methodology

The purpose of the study is to explore the possibility of shape generation conditioned on desired affordances. The main idea is to train a VAE to reconstruct voxelgrid object models, and then generate novel shapes by combining latent codes from existing examples with desired affordances.

The starting point for this research was the assumption that object affordances arise due to features that objects posses in relation to an actor (in this case a human user). Therefore, if we intend to create an object with desired affordances, it should possess corresponding features. The working hypotheses are: (i) objects providing the same affordance have common shape features, (ii) averaging over multiple shapes that provide the same affordance will extract a form providing that affordance, that we call *functional form*, (iii) interpolation between samples in the latent space can generate novel shapes providing the combined affordances of those samples. This last assumption is contentious, as we cannot yet predict the behavior of affordances when combining their underlying shapes. For this reason, we verify the presence of these affordances in simulation. We employ a fixed-size voxelgrid representation for all 3D object models, which has its benefits (easy to manipulate) and inconveniences (not a structure-aware representation). In what follows, *form* and *shape* are used interchangeably.

This paper continues by describing our workflow for object generation (section 3.1), and the *affordance arithmetics* operators used for generating shapes with desired affordances (section 3.2). We leave out the description of the neural network, as it is a standard Variational AutoEncoder with 3D Convolutional layers and a bottleneck latent layer (512 latent variables), taking as input voxelgrid models of dimension 64 × 64 × 64. It is trained on the ModelNet40 object dataset (Wu et al., 2015) using a weighted reconstruction loss, penalizing the network more strongly for errors in reconstructing full voxels. In terms of agents perceiving the affordances, all the objects in ModelNet40 were designed for humans. We employed the object categories that have a direct relationship between form and function (e.g., bathtubs, bowls, chairs, cups, desks, dressers, toilets), and excluded those for which this relationship was not present, considering that voxelgrid models have no moving parts (e.g., airplanes, cars, guitars, pianos, keyboards, radio). We also excluded the *person* category of models.

### 3.1. Proposed Workflow

Our workflow is composed of two phases: (1) learning phase, in which a neural network is trained to generate feature-based representations of objects and to faithfully reconstruct objects using this representation, and (2) request phase, in which a user requests the generation of a novel object with some desired affordances among those present in the training dataset of affordance-labeled objects. The algorithm then picks object categories providing those affordances, extracts the corresponding shape features (generating the form-to-function mapping), and combines them to generate a feature description of a new object. This description is then converted into a 3D voxelgrid model of the desired object.

The affordance labeling is done manually, with each object category in the dataset being assigned a list of affordances (using a CSV file). This assignment is done by the authors. In this context, multiple object categories can share the same affordance (e.g., sit-ability of chairs, sofas, toilets).

### 3.2. Operators for Manipulating Object Forms

In this section we describe the operators employed for manipulating object forms. First, we will describe the extraction of the *functional form* of an object category, which is the set of features that provides the affordances of that category. Second, we will describe how we combine two object descriptions into a single new one, which is expected to have the affordances of both input objects.

#### 3.2.1. Extract the Functional Form of an Object Category

Every category of objects possesses a set of affordances that defines it. From a *form follows function* perspective, all object samples contained in a category share a set of features that provide its set of affordances. We call this set of features the *functional form* of a category of objects. Multiple methods may exist for extracting it. For example, Larsen et al. (2016) isolated face features (e.g., presence of glasses, bangs, mustache) by computing the difference between the mean vector for categories with the attribute, and the mean vector for categories without the attribute. This was possible since all 2D images in the dataset belonged to a single category—*faces*, which were aligned and cropped. Unfortunately, this feature-extraction method is not applicable in our case, as there is no notion of alignment between the different categories of objects. In our particular case, we compute the vector of shape features that are responsible for the presence of affordances as the average latent vector of an object category. This *functional form* of an object category can then be visualized by inputting the obtained latent-vector description into the decoder trained to reconstruct 3D volumes. **Figure 6** illustrates some results obtained using this method.

Next, we assign an importance value to each latent variable composing the *functional form* of a category. We do this by computing the element-wise Kullback-Leibler (KL) divergence between the Probability Density Function (PDF) of these variables with the PDF of variables describing (1) a void volume, and (2) a non-informative distribution of independent Gaussians with zero mean and unit variance (called *prior*). The motivation behind using these two KL divergences for ranking the variables is to identify (1) which variables make the shape different from a void volume, to capture the filled voxels of the model, and (2) which variables distinguish the shape description from that of a Gaussian prior. Both of these KL divergences are normalized, so as to have unit norm. Then, an *importance vector* is defined as the weighted sum of the normalized KL divergences with a void and a prior distribution, with the corresponding weights *w*_*void*_ = 1/2 and *w*_*prior*_ = 1/2 chosen empirically.

#### 3.2.2. Combine Functional Forms of Two Object Categories

In order to combine two object descriptions (i.e., two latent vectors containing these descriptions), we need to identify which variables in each vector are important for encoding the object shape. In a degenerate case, if all the variables are critical for encoding the object shape, then their values cannot be changed, and therefore the object cannot be combined with another one (or a conflict resolution function must be devised). The hypothesis is that not all the variables are critical for representing the object shape, meaning that some variables' values can be neglected when combining two object descriptions. We identify which variables are important for an object description using the *importance vector* method described above.

The combination of two object descriptions is guided by their corresponding *importance vectors*. For simplicity, we describe the combination as being made between two object descriptions, although the method is applicable to any number of objects. A straightforward approach for generating a combined object description would be to overlay the combined feature vector onto a latent representation of a void space. This would leave out the irrelevant features of the functional forms employed in the combination. Such a combination would be commutative: the order in which objects are combined would not matter. However, although reasonable, this approach did not result in satisfactory outputs, the resulting models being mostly void. We hypothesize that it is because the samples from distinct categories are not spatially aligned, as compared to the example of face features taken from cropped and aligned face images (Larsen et al., 2016).

An alternative solution is the following. One object serves as a *base object*, from which are taken the initial values of the latent variables' distributions for the *combined object* description. The other object serves as *top object*, whose latent variables' distributions are combined with those of the *base object* according to the rules described in Table 1. The degree to which two object categories are combined can be controlled by varying the amount of information kept from each object description (i.e., the percentage of variables considered important for an object description). This combination operator is non-commutative, meaning that the combination of two objects can generate different results, depending on the order of objects in the combining operation.

**Table 1:** Interaction cases between latent variables contained in the descriptions of two different objects (*Obj*_*base*_, *Obj*_*top*_), which appear when attempting to combine them.

**#**	**Latent variable from Obj_*base*_**	**Latent variable from Obj_*top*_**	**Latent variable from Obj_*combined*_**
1	Non-important	Non-important	Value of base object
2	Non-important	Important	Value of important variable
3	Important	Non-important	Value of important variable
4	Important	Important	Average of the two values

We automatically generate a set of results, obtained using different thresholds for variable selection (0.5, 0.6, 0.7, 0.8, 0.95; one result per threshold value). The threshold value is a parameter of the algorithm, and is used as an additional degree of freedom, which allows the designer to put more emphasis on one affordance compared to the other.

Four cases appear when combining two latent vector descriptions of objects, as seen in Table 1. These rules can be resumed as follows: if both variable distributions are important then average them (case 4 in the table), if only one is important then keep the important one (cases 2 and 3), else keep the base values (case 1). **Figure 7** shows some outcomes of using these combination rules, including the impact of the order in which objects are combined, and of different threshold levels for the importance vectors (50, 60, 70, 80, and 95%).

## 4. Results and Discussion

In this section we provide our results on the (a) capacity of the VAE to describe and reconstruct objects, (b) extraction of functional forms for different object categories, (c) generation of novel objects through the combination of feature representations of object categories containing desired affordances, and (d) affordance testing for the generated objects. At the end of this section, we discuss limitations of the proposed method.

### 4.1. Object Representation and Reconstruction

**Figure 4:**
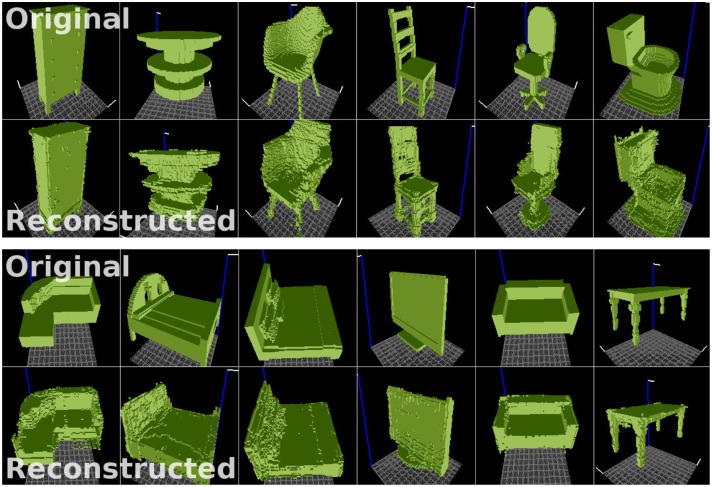
Examples of original voxelized objects **(top)** and their reconstructions **(bottom)** generated by the VAE. Objects taken from the ModelNet dataset (Wu et al., 2015). Visualizer: viewvox (Min, 2004).

**Figure 5:**
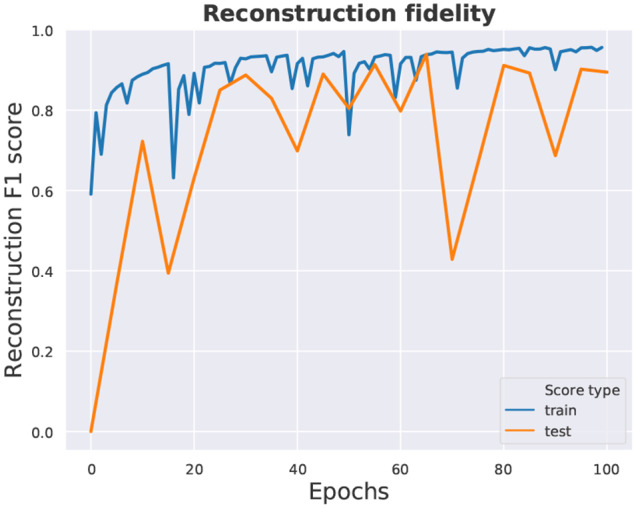
Reconstruction fidelity: F1 score on the train and test datasets. Since typically <10% of the voxelgrid volume is occupied by filled voxels in the training dataset (ModelNet40), a network that generates only empty volumes will have a ≈ 90% reconstruction score. Hence our use of weighting, valuing filled voxels proportionately more than empty ones.

Figure 4 illustrates 3D object samples and their corresponding reconstructions generated by the network. The satisfactory quality of reconstructions suggests that the encoder network can generate descriptions of objects in a feature (latent vector) space, and that the decoder network can successfully reconstruct objects from descriptions generated by the encoder. Our reconstructions are similar in quality to those shown in Wu et al. (2016), as we employ a similar 3D-convolutional architecture. Although we train on more object categories (30), the model has enough capacity to reconstruct samples from all categories relatively well. The reconstruction fidelity is reported in Figure 5.

### 4.2. Functional Form Extraction Results

**Figure 6:**
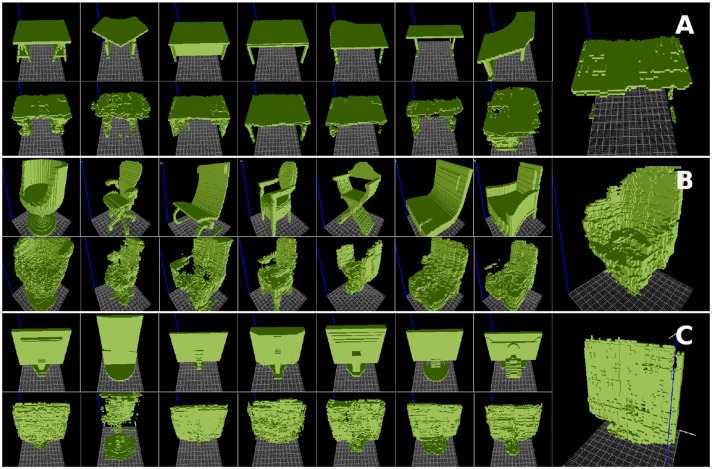
*Functional forms* extracted for **(A)** tables, **(B)** chairs, and **(C)** monitors taken from the ModelNet dataset (Wu et al., 2015). For each object category, the original samples are shown on top, the reconstructions on bottom, and the extracted functional form on the right.

Through the extraction of *functional forms* of different object categories, we expected to identify forms that provide affordances offered by those categories of objects. Figure 6 shows results on *functional form* extraction for tables, chairs, and monitors. Relevant features have been extracted, such as the flatness of tables providing *support-ability*, the seats and backrests of chairs providing *sit-ability* and *lean-ability*, and the flatness of monitors providing *display-ability*, respectively. Since supports differ in the selected tables, they are not in the set of common shape features (Figure 6A). In the chairs example, most selected samples had armrests, which led to this feature becoming part of the *functional form* (Figure 6B).

### 4.3. Object Combination Results

The ability to extract a shape representation that constitutes the *functional form* of a category, coupled with the ability to combine it with another object representation, makes it possible to extract and combine shape features that provide desired affordances.

#### 4.3.1. Sit-Ability and Contain-Ability

**Figure 7:**
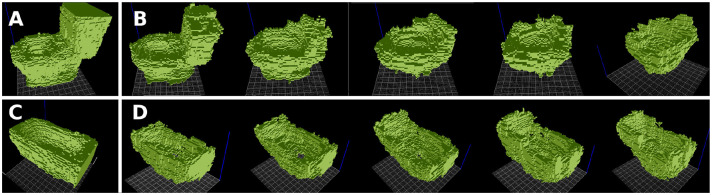
Combining features of *sit-ability* and *contain-ability*, extracted from toilets **(A)** and bathtubs **(C)**, into a novel object form, which can be interpreted as a bidet. **(B)** Toilet functional form with the bathtub functional form overlaid onto it. **(D)** Bathtub functional form with the toilet functional form overlaid onto it. A gradual transformation is displayed, combining the 50, 60, 70, 80, 95% of the functional forms. From left to right, the combination looks less like a toilet/bathtub, and more like a bidet.

**Figure 8:**
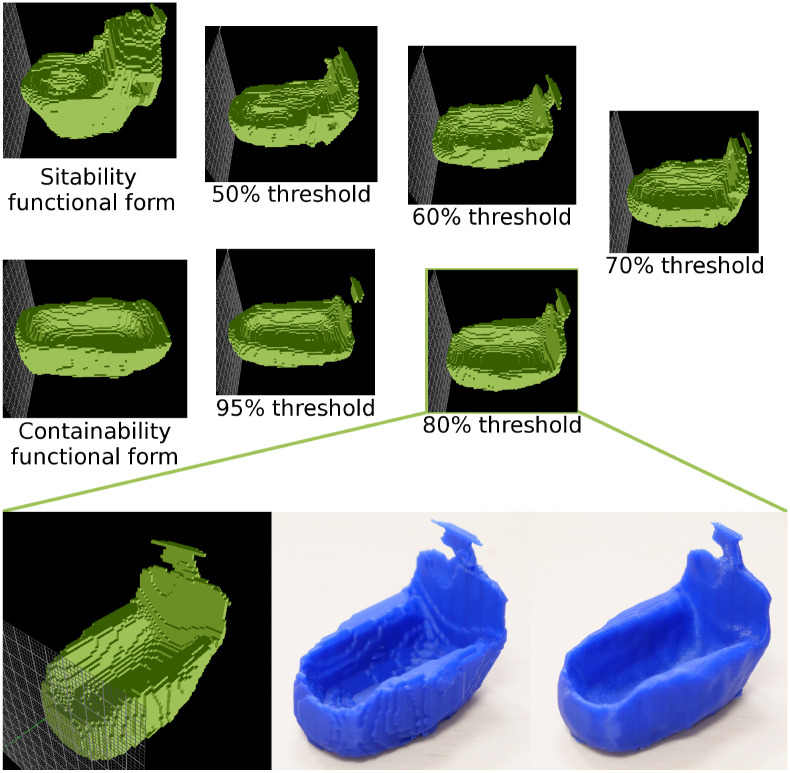
Another example of combining *sit-ability* of toilets with *contain-ability* of bathtubs. Below: the 3D printed versions (voxelized and smoothed) of the selected combination, obtained at 85% threshold.

In this experiment, we extract the *sit-ability* and *contain-ability* of toilet seats and bathtubs, respectively, in order to combine them into a new object providing both of these affordances. The obtained results may be interpreted as bidets (Figures 7, 8). For the extraction of functional forms, we used only aligned models within each object category (no rotations).

#### 4.3.2. Sit-Ability and Support-Ability

**Figure 9:**
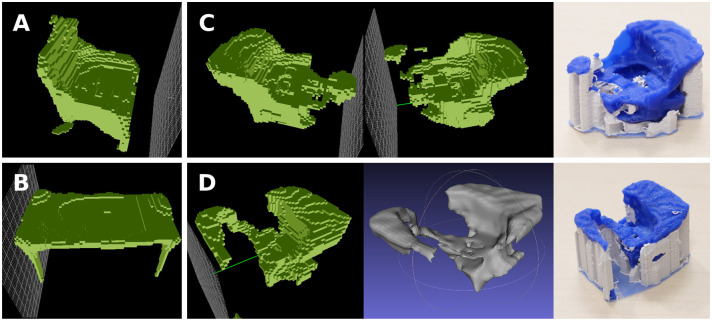
Combination of *sit*- and *support*- abilities from chairs and tables, which can be interpreted as a study chair. **(A)** Functional form of chairs. **(B)** Functional form of tables. **(C)** Combined object seen from different perspectives (threshold = 0.8). Note the flat surface in front of the chair, and also the flat surface on top of the backrest. On the right is the 3D printed shape in blue, with automatically generated support in white. **(D)** Combined object seen from different perspectives, and in 3D printed form (threshold = 0.7).

We combine the *sit* and *support* abilities of chairs and tables, respectively, in an attempt to recreate a study chair, which has a small elbow table. The result in Figure 9C is similar to the illustration in Figure 2. For the extraction of functional forms, we used only aligned models within each object category (no rotations).

#### 4.3.3. Contain-Ability and Support-Ability

**Figure 10:**
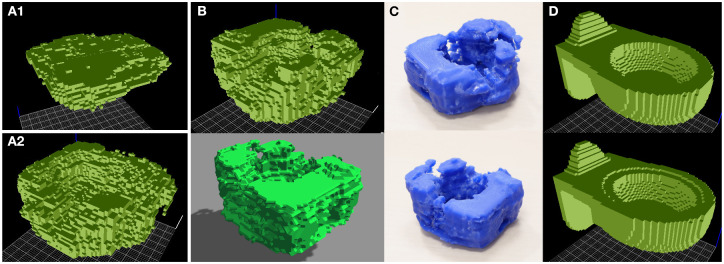
Combining features of *contain-ability* and *support-ability* into a novel object form providing both affordances. **(A1)** Table functional form, with its characteristic flatness providing *support-ability*. **(A2)** Bathtub functional form, providing *contain-ability* with its convex shape. **(B)** Bathtub shape with a flat surface on top, providing both *contain-ability* and *support-ability*, as seen in ViewVox (top) and Gazebo (bottom). **(C)** Smoothed, 3D printed version of this combined shape, seen from different angles. **(D)** Objects from the training set that are closest to the generated object, in terms of similarity of activation values in the one-before-last layer of the decoder.

This experiment displays the combination of *support-ability* and *contain-ability* affordances with the intent of creating something similar to a workdesk in a bathtub. The result is shown in Figures 10, 12. For the extraction of functional forms, we used the rotations of each object model by 0/90/180/270 degrees. This explains the difference between the functional forms of bathtubs and tables in Figures 7, 9 and those in Figure 10.

To ensure that the employed algorithm does not simply generate models by copying samples from the dataset, we compare the generated objects with the most similar samples from the dataset, based on the similarity of outputs of the one-before-last layer of the decoder. The result from Figure 10D suggests that generated objects are distinct from samples in the training set.

### 4.4. Affordance Confirmation Tests

We analyzed the generated objects using two methods: (1) verification of affordance presence using state-of-art affordance detectors, (2) testing affordance presence in a physics simulation. These are detailed below.

#### 4.4.1. Affordance Detectors

We attempted to identify the presence of the desired affordances (contain-ability, support-ability) using affordance detectors developed by other groups.

The affordance detector of Myers et al. (2015) was trained on real images, and does not generalize to synthetic images of objects in the Gazebo simulator. It failed to recognize the containability affordance in both standard (e.g., bowl, saucepan) and generated objects.

**Figure 11:**
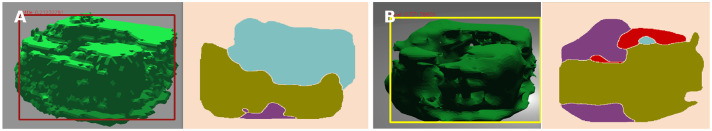
Affordance detection results using AffordanceNet (Do et al., 2018). **(A)** The AffordanceNet detector correctly identified *support-ability* (in light blue) and *wrap-grasp-ability* (in mustard color), and incorrectly identified *hit-ability* (in purple). **(B)** On a smoothed version of the object, and in different lighting conditions, AffordanceNet correctly identified *wrap-grasp-ability* (mustard), *contain-ability* (red), although with imperfect segmentation. It incorrectly identified *hit-ability* (purple) and *support-ability* (light blue).

We also tried the affordance detector of Do et al. (2018), called AffordanceNet. While it worked on objects viewed in simulation (including those of objects from the ModelNet40 dataset), it had difficulties with recognizing properly the affordances of generated objects (Figure 11A). We found experimentally that the failure cases for affordance detection were caused by the rugged surfaces of objects, and the fact that AffordanceNet was not trained on images of rugged objects. After applying Poisson smoothing to the surface of the object in Figure 11A, the detector correctly identified the presence of *contain-ability*, although it still struggled to locate it properly (Figure 11B).

#### 4.4.2. Affordance Testing in Simulation

To verify that the generated objects indeed provide the requested affordances, we developed *affordance tests* to execute in simulation. For this purpose, the generated voxelgrid model is converted into a mesh using the *marching cubes* method (Lorensen and Cline, 1987), after which we compute its inertia matrix and create the Spatial Data File (SDF) for importing it into the Gazebo simulator (Koenig and Howard, 2004) with a Bullet physics engine. These computations are made using the Trimesh (Dawson-Haggerty et al., 2019) Python library. All the objects in employed ModelNet40 dataset were made for humans, and therefore implicitly consider humans as actors interacting with these objects. They also contain the human-designer bias.

**Figure 12:**
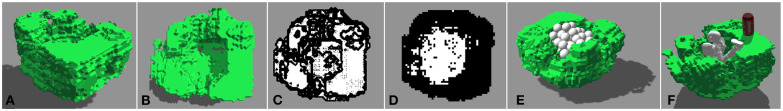
The generated object with contain-ability and support-ability (similar to a bathtub-workdesk, or *bathdesk*) in **(A)** perspective view and **(B)** top-down view. **(C)** Result of the support-ability test: white pixels show locations with support-ability. **(D)** Contain-ability map. **(E)** Still frame from the contain-ability test with spheres. **(F)** Demonstration that the workdesk-bathtub can fit an iCub humanoid robot, and support a tin can.

**Figure 13:**
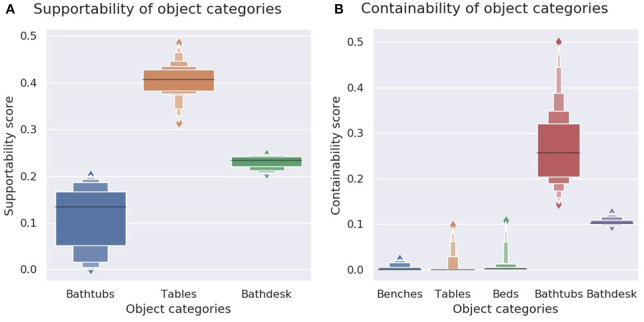
**(A)** Support-ability scores for bathtubs, tables, and generated *bathdesk* objects. These afford support-ability, albeit to a lesser degree than tables. **(B)** Contain-ability scores for bathtubs, beds, benches, tables (a dozen samples from each category), and of generated *bathdesk* objects.

To verify for supportability, we suspend the object (to remove influence from the object bottom shape) and verify which of its regions can support a stable object with a flat base, by dropping from above from different (x,y) locations a cube with mass 1 kg and a down-oriented flat face, and checking how this influences the (x,y) coordinates of the cube centroid. If only its z coordinate (altitude from ground) changes, while the (x,y) coordinates remain the same (meaning the cube landed flat on the object) then that location is marked as providing stability. On the contrary, if the object region below the cube is not flat, the cube tumbles over, landing on (x,y) coordinates distinct from the initial ones. If the cube missed the object and landed on the floor directly below its drop coordinates, then this location is marked as empty. Since we are looking to reproduce the support functionality of tables, we only consider the support-ability of surfaces above the object centroid. Without this criterion, bathtubs with a flat bottom score high in the support-ability evaluation. Figure 12C shows the calculated support-ability map of the generated object, while Figure 13A shows the computed support-ability scores for a dozen of tables, bathtubs, and their functional combination. A video of a support-ability test can be seen in the Supplementary Material^1^.

To verify contain-ability, we pour spheres into the object until they overflow and fall on the ground, and measure the ratio of the total volume of spheres contained inside the object vs. the volume of object's convex hull (Figure 12E). A video of a contain-ability test can be seen in the Supplementary Material of this paper^2^.

This allows to compare the *contain-ability* of two different objects. Figure 13B shows the contain-ability measurements for some ModelNet40 object samples (≈ 12 objects per category).

We also compute a 2D contain-ability map of the object (Figure 12D), which highlights the regions capable of containing an object, preventing it from touching the ground. To compute this map, the procedure is as follows: (1) Discretize the plane on top of the object using a grid, (2) At each discretized location: (2a) Identify if there is an object below, by dropping a ball and observing if it lands on the ground directly below it (same x,y coordinates), (2b) If there is an object below, identify if this object location provides contain-ability by repeatedly dropping a sphere with different angular velocity (roll, pitch) and observing if it always ends above the ground (inside the object) after a given time (*T* = 12 real time seconds).

We observe from the measurements in Figure 13 that the generated object manages to retain affordances from both its parent objects, but with a trade-off in its performance.

### 4.5. Limitations

The proposed method currently has a set of limitations:

1. **Intra-category alignment:** The method used for extracting functional forms from object categories, which employs *averaging* the gaussians describing the voxel locations, requires all samples inside an object category to be aligned. We intend to remove this requirement by aligning samples using the method proposed by Suwajanakorn et al. (2018).
2. **Inter-category alignment:** The current setup (voxelgrid representation, feature transfer in latent representation space) allows transfer of shape features only between aligned object forms. While the concept of alignment is meaningful for objects within a single category, it is (arguably) meaningless for objects of different categories (e.g., alignment of spoons with sofas, monitors, screens with curtains).
3. **Combination feasibility:** The combination method does not state if a solution to the posed problem does not exist (i.e., if combining two different sets of affordances is possible, like *stability* and *rollability*).
4. **Scale variation:** The different scales of objects are not considered when combining objects. Training the neural network on object models which are correctly sized relative to each other would solve this issue. A naïve solution would be to increase the size of the input voxel cube, to fit inside detailed descriptions of both small-scale objects (e.g., spoons, forks, chairs) and large scale objects (e.g., dressers, sofas), which would also increase the training time.
5. **Shape representation:** Voxelgrids, like meshes and point clouds, are a low-level representation of shapes. Training deep learning models to encode high-resolution voxelgrids requires a high memory and time complexity. Moreover, voxelgrids are incapable to encode curved shapes. In addition, not all voxegrid states correspond to valid shapes, as they can contain more than one connected component.
6. **Shape centering:** Since we trained the network on samples centered in the voxelgrid volume, the features describe mostly voxels in the center of the bounding cube. This aligns shapes by their centroid location, allowing easy intra-category alignment of samples. However, centroid alignment is not always a correct alignment for transferring and combining shape features. Thus, combining two different feature descriptions makes them compete for the center voxels in this bounding volume. Introducing an operator for spatially offsetting some shape features would allow to construct composite objects.

## 5. Conclusion and Future Work

We have presented a method for generating voxelgrid objects with desired affordances, by first extracting a form-to-function mapping from a dataset of objects, and then manipulating and combining these forms through *affordance arithmetic*. The method relies on a neural network Variational Auto-Encoder to extract feature-based descriptions of objects. These descriptions allow shape manipulation and shape arithmetics in a latent feature space, before being transformed back into 3D voxelgrid models. We then test the presence of desired affordances in a physical simulator and with an affordance detector.

In contrast to an ontology-based approach, where modifications can be done deterministically, all object shape manipulations are probabilistic in our case. Thus, generated inexact models serve only as *design suggestions*. However, a production-grade technology would require less noisy object-modeling results. We are currently investigating the possibility of incorporating a GAN-style discriminator (Wu et al., 2016) in our framework in order to encourage the generation of objects with smooth surfaces similar to those of existing man-made objects. However, due to the inherent instability of Generative Adversarial Network (GAN) training, our results currently do not show an improvement over the proposed VAE-only architecture. We also plan to implement a training procedure to encourage neurons in the latent layer to represent specific transformations (rotation, scale) following the approach of Kulkarni et al. (2015).

Our models still lack information about materials from which objects are composed, their colors or textures, and the articulations between subparts. Adding it would make the approach much more practical. Using a deterministic and interpretable object structure representation (Tian et al., 2019) may also constrain and simplify the shape generation problem.

The voxelgrid representation comes with a trade-off (easy to use, but low model resolution, high computational complexity for training the neural network), and it is possible to revise this decision in our future work. This would require considering alternative low-level representations such as point clouds, mesh representations, or high-level procedural, structural, hierarchical representations. The difficulty of combining features from different object categories caused by the absence of spatial alignment between objects suggests that spatial descriptions (like voxelgrids) are not well-adapted for generating shapes combining multiple features. Thus, representations such as *Constructive solid geometry* (Foley et al., 1996) that define a shape as a sequence of modifications applied to an existing solid part, may be better adapted for shape generation.

In addition, instead of using a dataset containing an implicit mapping of form-to-function (with affordance-labeled object categories), we intend to learn object affordances automatically, by letting a robot interact autonomously with a set of objects. This is related to the currently active field of *affordance learning* in robotics. Moreover, the use of 3D shape descriptors developed in this research will facilitate affordance learning and knowledge transfer for autonomous robots.

In the future, models of furniture with specific functionalities generated by our algorithm could be assembled in the physical world using re-configurable modular robots (Khodr et al., 2019). This would allow the creation of custom shapes for transformer-furniture.

## Data Availability Statement

The generated models are publicly available at https://gitlab.com/mandries/generated-objects. The source code will be published at https://gitlab.com/mandries/shape-generation-voxelgrid.

## Author Contributions

Literature review by MA. Methodology and theoretical developments by MA and AD. Experiment design and implementation by MA and AD. Analysis of the experimental results by MA, AD, and JS-V. Document writing and illustrations by MA and AD. All authors contributed to manuscript revision, read, and approved the submitted version.

## Conflict of Interest

The authors declare that the research was conducted in the absence of any commercial or financial relationships that could be construed as a potential conflict of interest.

## Footnotes

^1^Support-ability test: https://www.youtube.com/watch?v=jnKjk5eQ_dg.

^2^Contain-ability test: https://www.youtube.com/watch?v=GbvFFesxUrQ.
