# Supplementary figures and images for: Automatic Generation of Object Shapes With Desired Affordances Using Voxelgrid Representation
Source: Front Neurorobot. 2020 May 14;14:22. doi: 10.3389/fnbot.2020.00022 (PMC7240024; doi:10.3389/fnbot.2020.00022)

# Supportability of object categories

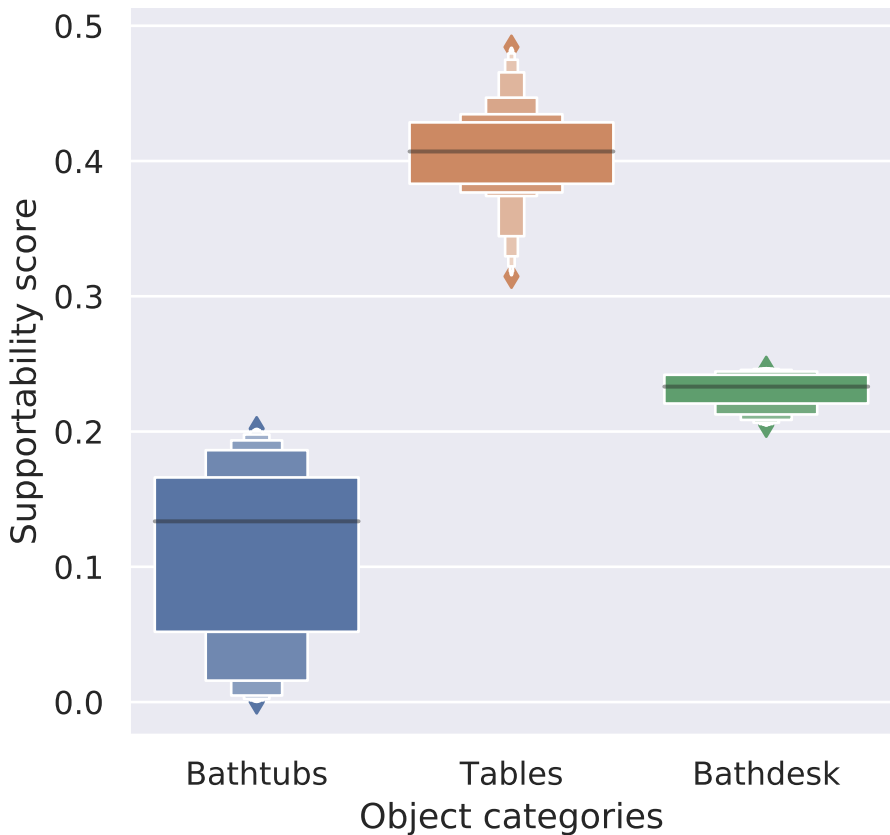

Supplement: Supplementary file 3 [file Data_Sheet_1.PDF]

# Containability of object categories

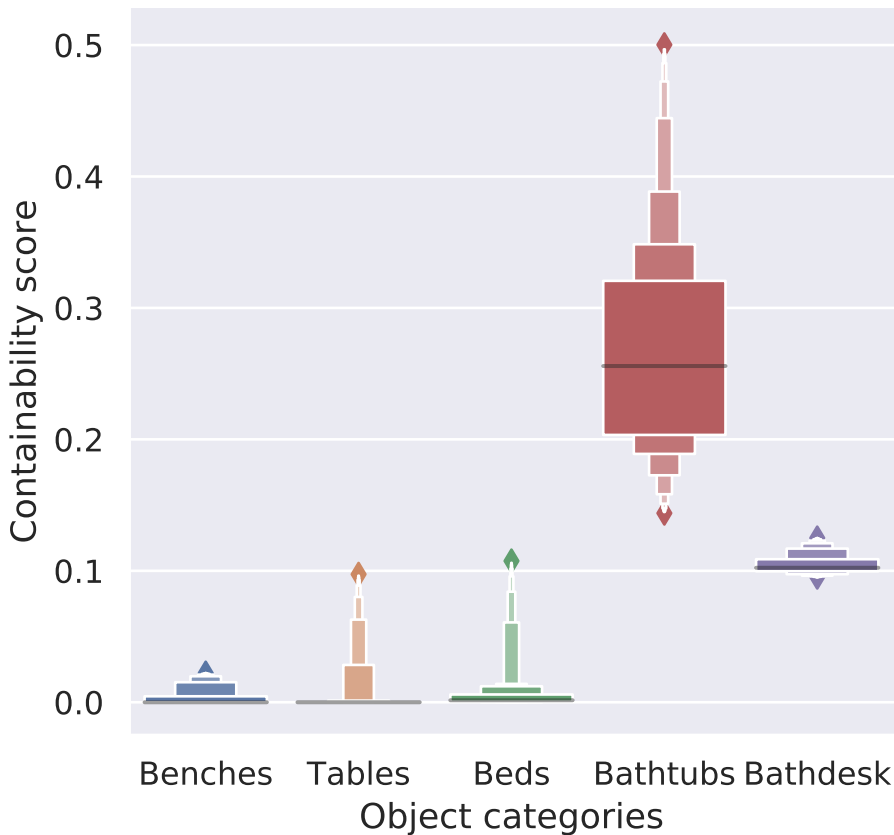

Supplement: Supplementary file 4 [file Data_Sheet_2.PDF]
